# Capsule-Targeting Depolymerases Derived from *Acinetobacter baumannii* Prophage Regions

**DOI:** 10.3390/ijms23094971

**Published:** 2022-04-29

**Authors:** Alena Y. Drobiazko, Anastasia A. Kasimova, Peter V. Evseev, Mikhail M. Shneider, Evgeniy I. Klimuk, Alexander S. Shashkov, Andrei S. Dmitrenok, Alexander O. Chizhov, Pavel V. Slukin, Yuriy P. Skryabin, Nikolay V. Volozhantsev, Konstantin A. Miroshnikov, Yuriy A. Knirel, Anastasia V. Popova

**Affiliations:** 1 Moscow Institute of Physics and Technology (National Research University), 141700 Dolgoprudny, Russia; drobyazko@phystech.edu; 2 Center of Life Sciences, Skolkovo Institute of Science and Technology, 121205 Skolkovo, Russia; 3 N. D. Zelinsky Institute of Organic Chemistry, Russian Academy of Sciences, 119991 Moscow, Russia; nastia-kasimova979797@mail.ru (A.A.K.); shash@ioc.ac.ru (A.S.S.); dmt@ioc.ac.ru (A.S.D.); chizhov@ioc.ac.ru (A.O.C.); yknirel@gmail.com (Y.A.K.); 4 Shemyakin-Ovchinnikov Institute of Bioorganic Chemistry, Russian Academy of Sciences, 117997 Moscow, Russia; petevseev@gmail.com (P.V.E.); mikhailshneider@gmail.com (M.M.S.); kmi@bk.ru (K.A.M.); 5 Institute of Molecular Genetics of National Research Centre «Kurchatov Institute», 123182 Moscow, Russia; jonikl@gmail.com; 6 State Research Center for Applied Microbiology and Biotechnology, 142279 Obolensk, Russia; xopgi@yandex.ru (P.V.S.); sjurikp@gmail.com (Y.P.S.); nikvol@obolensk.org (N.V.V.)

**Keywords:** *Acinetobacter baumannii*, prophages, phage receptor-binding proteins, structural depolymerase, glycosidase, capsular polysaccharide, capsular type

## Abstract

In this study, several different depolymerases encoded in the prophage regions of *Acinetobacter baumannii* genomes have been bioinformatically predicted and recombinantly produced. The identified depolymerases possessed multi-domain structures and were identical or closely homologous to various proteins encoded in other *A. baumannii* genomes. This means that prophage-derived depolymerases are widespread, and different bacterial genomes can be the source of proteins with polysaccharide-degrading activities. For two depolymerases, the specificity to capsular polysaccharides (CPSs) of *A. baumannii* belonging to K1 and K92 capsular types (K types) was determined. The data obtained showed that the prophage-derived depolymerases were glycosidases that cleaved the *A. baumannii* CPSs by the hydrolytic mechanism to yield monomers and oligomers of the K units. The recombinant proteins with established enzymatic activity significantly reduced the mortality of *Galleria mellonella* larvae infected with *A. baumannii* of K1 and K92 capsular types. Therefore, these enzymes can be considered as suitable candidates for the development of new antibacterials against corresponding *A. baumannii* K types.

## 1. Introduction

*Acinetobacter baumannii* is a nosocomial pathogen that causes pneumonia, wound and catheter-related urinary tract infections, peritonitis, meningitis, endocarditis, and bloodstream infections [1]. Due to the growing prevalence of isolates with limited treatment options, *A. baumannii* has been listed by the World Health Organization as a critical priority pathogen for therapeutics development [2]. Capsular polysaccharide (CPS) is one of the essential *A. baumannii* virulence determinants composed of many oligosaccharide repeats (K units), which forms a thick protective layer around the bacterial cell. The polymorphism of the chromosomal capsule loci (K loci, KL) is responsible for the observed high diversity of *A. baumannii* CPS structures (more than 144 K types have now been identified) [3,4,5].

Genomes of many lytic bacteriophages contain genes encoding proteins with polysaccharide-degrading activities [6]. These are highly specific structural depolymerases combining the functions of the cleavage of capsular polysaccharides and phage attachment to the bacterial host cell.

We suggest that the genetic material of temperate phages integrated in bacterial genomes (or prophages) can also contain genes encoding different receptor-binding proteins (RBPs), including structural depolymerases. During an analysis of several *A. baumannii* genomes deposited in Genbank, we identified open reading frames (ORFs) encoding proteins with the bioinformatically predicted polysaccharide-degrading activities and structural similarities to tailspike depolymerases of different virulent phages. Considering the high prevalence of prophages in *A. baumannii* genomes [7,8], we assumed that genetic material of prophages could be a source of different polysaccharide-degrading enzymes. The use of these prophage-derived depolymerases, such as the described specific phage depolymerases, could be an effective approach to control bacterial cells surrounded by polysaccharide capsules and the extracellular polymer matrix of biofilms.

The aim of this work was to characterize several different prophage-derived depolymerases encoded in *A. baumannii* genomes. For two of studied depolymerases, designated as DpoAB5075 and DpoB8300, we determined the specificity towards CPSs of *A. baumannii* K1 and K92 capsular types, respectively, and elucidated the mechanisms of their specific action. We demonstrated that recombinant proteins with established enzymatic activity significantly reduce the mortality of *Galleria mellonella* larvae infected with *A. baumannii* of corresponding K types. Thus, in our opinion, prophage-derived depolymerases degrading *A. baumannii* CPS of a certain structure can be considered as effective antivirulence agents as well as described lytic phage-derived enzymes.

## 2. Results

### 2.1. Identification of Prophage-Derived Depolymerases

**Figure 1:**
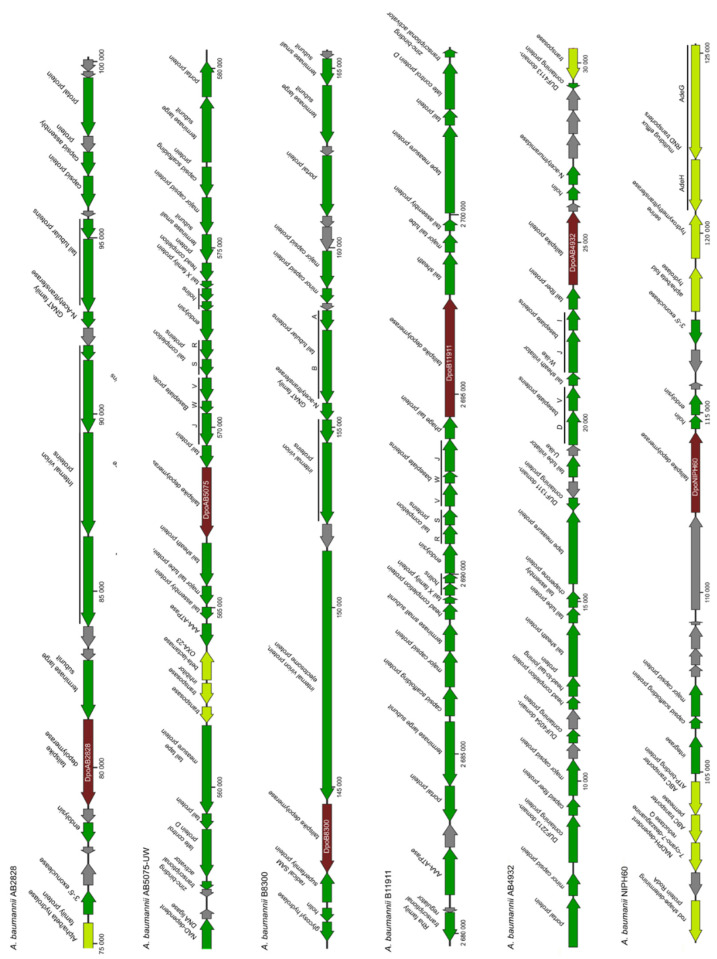
Genetic maps of *A. baumannii* genome fragments (approximately 25,000 base pairs) containing determined prophage-derived genes. ORFs are indicated as arrows, the direction of an arrow shows the direction of transcription. The names of *A. baumannii* strains, where the genes encoding structural depolymerases have been found, are shown above the genetic maps. The ORFs were validated and curated using a search in the non-redundant database of the NCBI and HHpred profile–profile search. The ORFs are coloured according to the following predictions: green for prophage-derived genes encoding phage assembly and structural proteins, products involved in phage nucleotide metabolism, packaging of DNA into the capsid, bacterial cell lysis, and integration of phage DNA into the bacterial genomes; dark red for the genes encoding predicted structural or tailspike depolymerases, conditionally designated as DpoAB2828, DpoAB5075, DpoB8300, DpoB11911, DpoAB4932, and DpoNIPH60; gray for genes of hypothetical proteins; light green for genes responsible for the functioning of the bacterial cells. Maps were created using SnapGene software (from Insightful Science; available at snapgene.com, accessed on 12 January 2022).

For our analysis, we randomly chose several genome sequences of *A. baumannii* strains isolated from different geographical regions. We identified the coordinates of possible prophage regions (complete or truncated/incomplete prophage genomes) in these sequences using the PHASTER [9]. After that, we reannotated some of predicted prophage regions and adjacent areas of the *A. baumannii* genomes using a search in the NR (non-redundant) database of the NCBI [10] and HHpred profile–profile search [11]. These regions were confirmed to contain genes encoding phage assembly and structural proteins, products involved in phage nucleotide metabolism, the packaging of DNA into the capsid, bacterial cell lysis, and the integration of phage DNA into the bacterial genomes. The fragments of *A. baumannii* genomes containing determined prophage-derived genes are presented in Figure 1.

**Figure 2:**
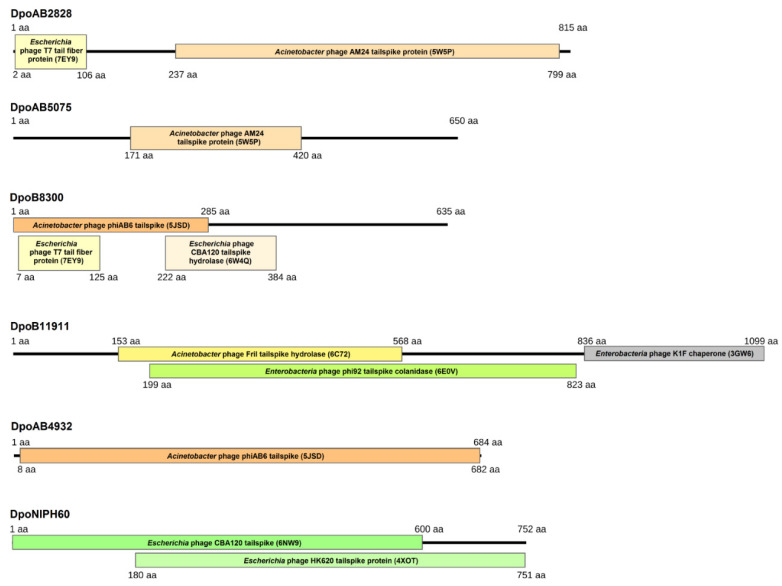
HHpred-detected structural similarities between prophage-derived depolymerases and proteins from the PDB databases.

Several ORFs encoding proteins with predicted polysaccharide-degrading activities or those that shared homology with different phage RBPs (tail fibers or tailspikes) were identified in some determined prophage regions. We chose six genes located in the genomes/genome contigs of *A. baumannii* strains AB2828, AB5075-UW, B8300, B11911, AB4932, NIPH60 (Table 1). The proteins encoded by these genes were conventionally named according to the designations of corresponding *A. baumannii* strains. For example, the depolymerase encoded in the prophage region of *A. baumannii* B8300 genome (CP021347) was conventionally designated as DpoB8300, etc. Since the proteins, DpoAB2828, DpoAB5075, DpoB8300, DpoB11911, DpoAB4932, and DpoNIPH60, were found to be structurally similar to tailspikes of different virulent phages (Figure 2), they can all be assigned as prophage-derived structural depolymerases, most likely prophage tailspike depolymerases. In all cases, predicted depolymerases were surrounded by other prophage-derived genes, such as different tail and baseplate proteins, major capsid proteins, terminase and portal proteins, endolysins and holins, integrases, etc. (Figure 1).

BLASTp analysis revealed that predicted depolymerases DpoAB2828, DpoAB5075, DpoB8300, DpoB11911, DpoAB4932, and DpoNIPH60 were completely identical to different proteins encoded in various *A. baumannii* genomes (GenBank: WP_002009999, WP_000729646, WP_049590628, WP_053093879, WP_000224779, and WP_004840621, respectively). 

Depolymerase DpoAB2828 was also found to share similarity with the tailspike protein gp48 of lytic *Acinetobacter* phage APK09 (UAW09804, the coverage obtained to an E-value of 6 × 10^−173^ was 62% with an identity of 52%) and tailspike protein gp45 of lytic *Acinetobacter* phage vB_AbaP_B1 (YP_009610331, the coverage obtained by an E-value of 1 × 10^−171^ was 70% with an identity of 47.29%) belonging to the family *Autographiviridae*, the genus *Friunavirus*. Depolymerase DpoAB4932 was completely identical to the tail fibre protein of temperate *Acinetobacter* phage Ab105-1phi (ALJ99087) and almost identical to the tail fibre protein of temperate *Acinetobacter* phage AbTJ (QAU04146, the coverage obtained by an E-value of 4 × 10^−165^ was 97% with an identity of 96.26%) belonging to the family *Myoviridae.* Depolymerase DpoNIPH60 shared a similarity to the tailspike protein gp79 of lytic *Acinetobacter* phage TaPaz (QVW53860, the coverage obtained by an E-value of 0.0 was 86% with an identity of 49.77%) and the tailspike protein gp43 of lytic *Acinetobacter* phage Cato (UMO77867, the coverage obtained by an E-value of 4 × 10^−165^ was 84% with an identity of 44.32%) assigned to the family *Myoviridae*.

According to HHpred analysis, the N-terminal parts of DpoAB2828 and DpoB8300 shared a structural similarity with the N-terminal part of *Escherichia* phage T7 tail fiber protein gp17. The remaining parts of all analyzed depolymerases contained regions that showed structural similarities with different phage carbohydrate-hydrolyzing enzymes or tailspikes (Figure 2). The C-terminal domain structure of DpoB11911 was predicted as an intramolecular chaperone, which most likely participated in correct protein folding [12]. Similar structures were located at the C-termini of *A. baumannii* phage vB_AbaP_AS12 tailspike depolymerase AS12_gp42 (PDB ID: 6EU4) and tail proteins of *E. coli* phage K1F (PDB ID: 3GW6), *E. coli* phage T5 (PDB ID: 4UW8), and *Bacillus* virus GA-1 (PDB ID: 3GUD).

### 2.2. Prediction of the Domain Organization of Prophage-Derived Depolymerase Monomers

**Figure 3:**
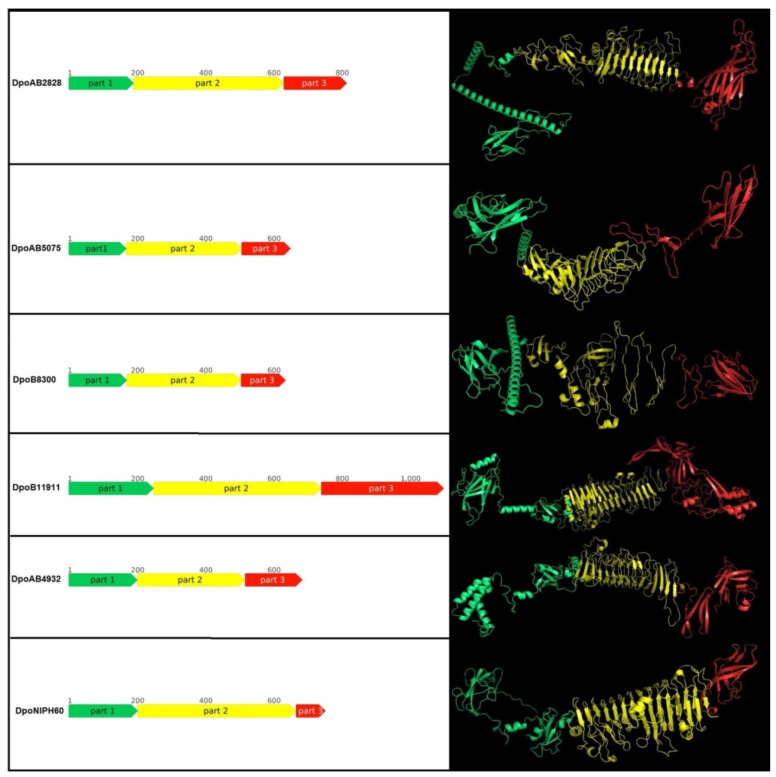
Schematic representation and predicted spatial structure of the prophage-derived depolymerases. The parts of proteins are coloured according to their suggested roles. Part 1 (coloured green) contains the particle-binding N-terminal domain, part 2 (coloured yellow) contains the central pyramidal domain, the part 3 (coloured red) contains C-terminal domain.

The domain structures of prophage-derived depolymerase monomers predicted with AlphaFold 2 [13] were shown to include several regions, conventionally designated as part 1, part 2, and part 3 (Figure 3).

Part 1 of depolymerase monomers possesses a complex structure. This part can be responsible for the attachment of these proteins to the phage particles. The predicted structures of part 1 for DpoAB2828, DpoAB5075, DpoB8300, DpoB11911, and DpoNIPH60 essentially include an antiparallel β-sheet structured N-terminal part followed by α-helices. The modelled structure of DpoAB4932 part 1 was predicted to include an α-helical part comprising roughly the first 100 N-terminal amino acid residues followed by the antiparallel β-sheet structure.

Except for the DpoB8300 predicted structure, all other prophage-derived structural depolymerases contain a parallel β-structured pyramidal central part, which can form the “pyramid domain” (part 2) [14,15] upon trimerization. The central part of the predicted DpoB8300 structure also seems to include a pyramid-like region, but the secondary structure of this region was not determined. Most likely, it was impossible to make a better prediction with AlphaFold due to the lack of resolved structures of similar sequences.

Parts 3 of all the modelled depolymerases includes a C-terminal antiparallel β-structured region of about 100 amino acids. It seems that the structure of this region is reminiscent of the structure of the receptor-binding, carboxy-terminal domain of phage T7 tail fiber, which forms a β-sandwich containing two sheets of four β-strands each. Part 3 of a longer protein DpoB11911 also includes additional structures comprising α-helices and β-sheets, most likely corresponding to predicted intramolecular chaperone.

Approximate positions of the central part (part 2), N-terminal part to the right of the central domain (labelled as part 1) and C-terminal part to the left of the central domain (labelled as part 3) are shown in Figure 3.

### 2.3. The Phylogenetic Analysis

**Figure 4:**
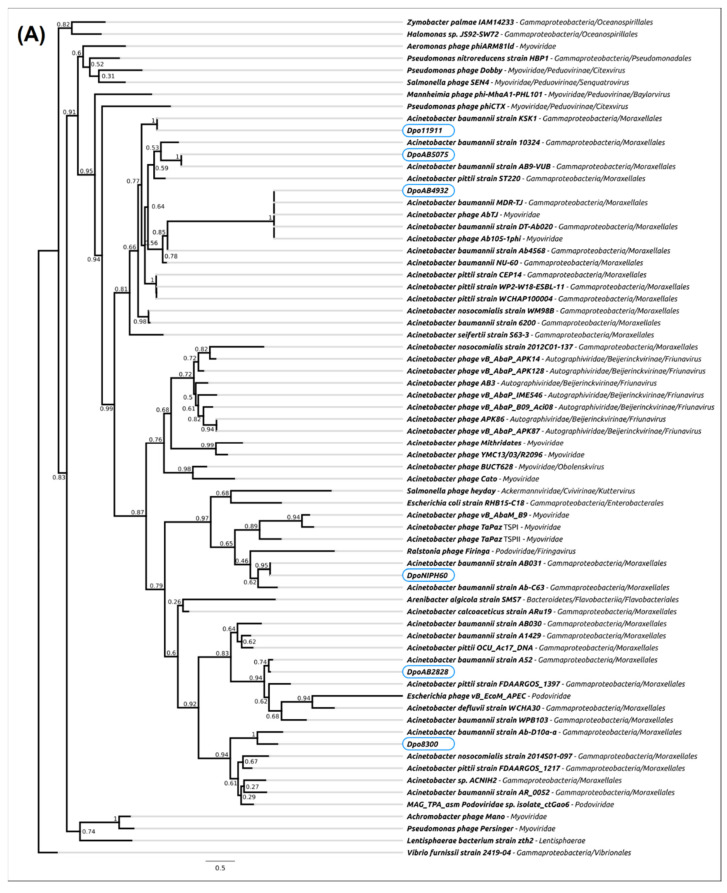

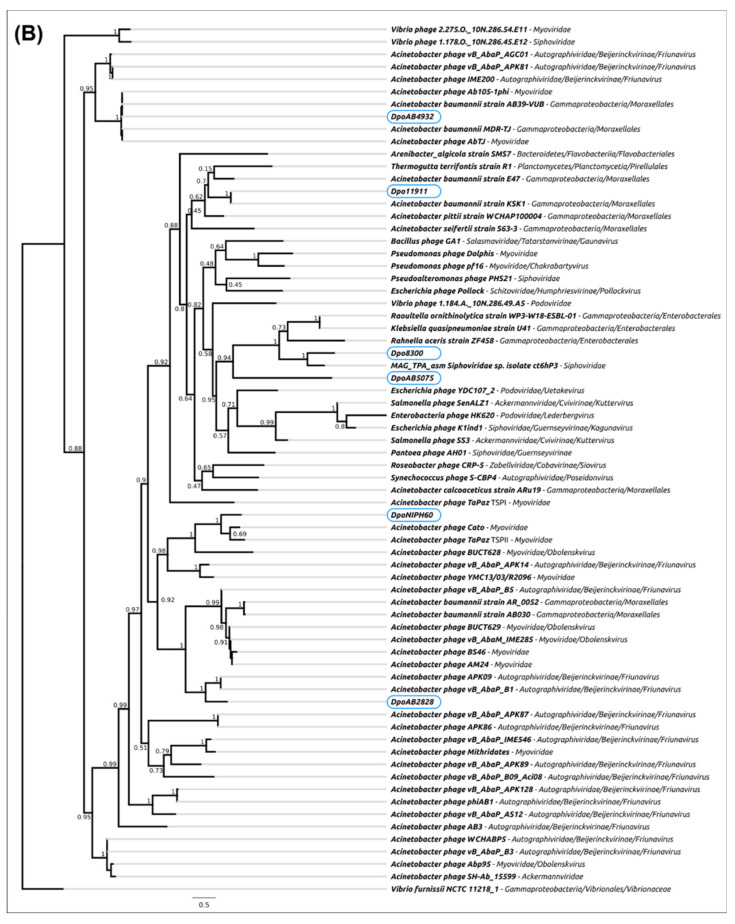
Best-scoring phylogenetic trees constructed with RAxML-NG based on the protein sequences of the studied prophage-derived depolymerases and homologous sequences found in NCBI nr/nt database. (**A**) The tree built with the N-terminal parts of the proteins (part 1 in Figure 3). (**B**) The tree built with CPS-recognizing/degrading parts of the proteins (combined parts 2 and 3 in Figure 3). Bootstrap support values are shown above their branch as a share of 1000 replicates. The scale bar shows 0.5 estimated substitutions per site. *Vibrio furnissii* NCTC 11218_1 was used as an outgroup.

A phylogenetic analysis was conducted using amino acid sequences of N-terminal parts (parts 1 in Figure 3) and CPS-recognizing/degrading parts (combined parts 2 and 3 in Figure 3) of the prophage-derived depolymerases and homologous sequences found with the BLAST search (Figure 4A,B).

The results of the phylogenetic analysis of N-terminal domains responsible for binding to phage particles (Figure 4A) showed that these parts of prophage-derived depolymerases were most closely related to the corresponding parts of different proteins encoded in various *A. baumannii* genomes. Since depolymerases are structural proteins, the similarity of amino acid sequences of their N-terminal domains to corresponding parts of other proteins most likely reflect the fact that temperate phages carrying these proteins could be representatives of closely related taxonomic groups.

The results of the phylogenetic analysis of CPS-recognizing/degrading parts of the prophage-derived depolymerases (Figure 4B) showed that these parts were also related to different *A. baumannii* proteins, most likely of prophage origin. Moreover, depolymerase Dpo2828 formed a distinct monophyletic branch with related proteins encoded in genomes of *Friunavirus* phages APK09 and vB_AbaP_B1, and depolymerase DpoNIPH60 was related to the proteins encoded in genomes of lytic phages TaPaz and Cato, belonging to the family *Myoviridae*.

In both phylogenetic analyses, depolymerase DpoAB4932 was found to be very closely related to the proteins encoded in the genomes of temperate phages Ab105-1phi and AbTJ assigned to the family *Myoviridae*. This means that all these proteins could probably be specific towards CPS with a certain structure. However, this suggestion requires further experimental confirmation.

Interestingly, DpoB8300 was phylogenetically related to the proteins encoded in a siphoviral phage and *Klebsiella* bacterial genomes, reflecting the possible results of horizontal transfers between bacteria belonging to comparatively distant taxa sharing the same ecological niche.

Differences in the topologies of the trees built using different parts of prophage-derived depolymerases indicate the possibility of the independent evolution of different domains of phage receptor-binding proteins.

### 2.4. The K-Specificity of Prophage- Derived Depolymerases

Deletion mutants lacking the N-terminal domains of the prophage-derived depolymerases were cloned, expressed, and purified (Figure S1). The described expression conditions and a combination of metal affinity and gel permeation chromatography yielded approximately 10–15 mg of active proteins from 1 L cell culture.

**Figure 5:**
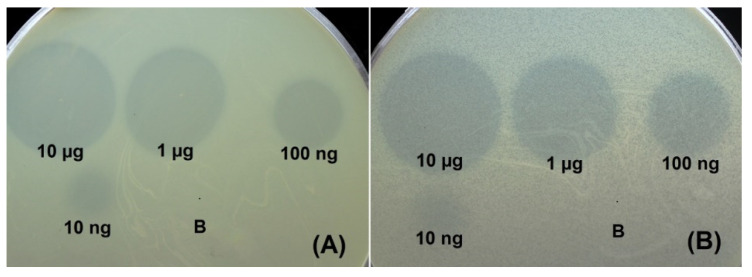
Spot test with serial 10-fold titration of purified recombinant depolymerases DpoB8300 (**A**) and DpoAB5075 (**B**) and on *A. baumannii* B8300 and AYE lawns, respectively, after 18 h of incubation; B—buffer (20 mM Tris-HCl, pH 7.5, and 150 mM NaCl) for storage of the proteins as a negative control.

The specificity of prophage-derived depolymerases, DpoAB2828, DpoAB5075, DpoB8300, DpoB11911, DpoAB4932, and DpoNIPH60, was tested against a collection of *A. baumannii* strains belonging to 56 different capsular type (Table S1). Two of six recombinant proteins expressed in *E. coli* exhibited depolymerase activities on the bacterial lawns of *A. baumannii* belonging to different capsular types. Namely, the depolymerase DpoAB5075 was specific to CPS of *A. baumannii* AYE (capsular type K1), and the depolymearse DpoB8300 was specific to CPS of *A. baumannii* B8300 (K92). These depolymerases formed opaque haloes on the bacterial lawns of *A. baumannii* strains of the corresponding K types. An example of a serial 10-fold titration of the purified recombinant depolymerases on the bacterial lawns of *A. baumannii* strains, after 18 h of incubation, is presented in Figure 5. The proteins were stable for at least 3 months at 4 °C, retaining sufficient depolymerase activities.

For the remaining depolymerases, which were cloned, expressed and purified, we were unable to identify specificity to any of the tested K types.

### 2.5. Mechanisms of Cleavage of A. baumannii CPSs by Prophage-Derived Depolymerases

To elucidate the mechanisms of action of prophage-derived depolymerases, DpoAB5075 and DpoB8300, the CPSs of *A. baumannii* AYE and B8300 were isolated and purified.

**Figure 6:**
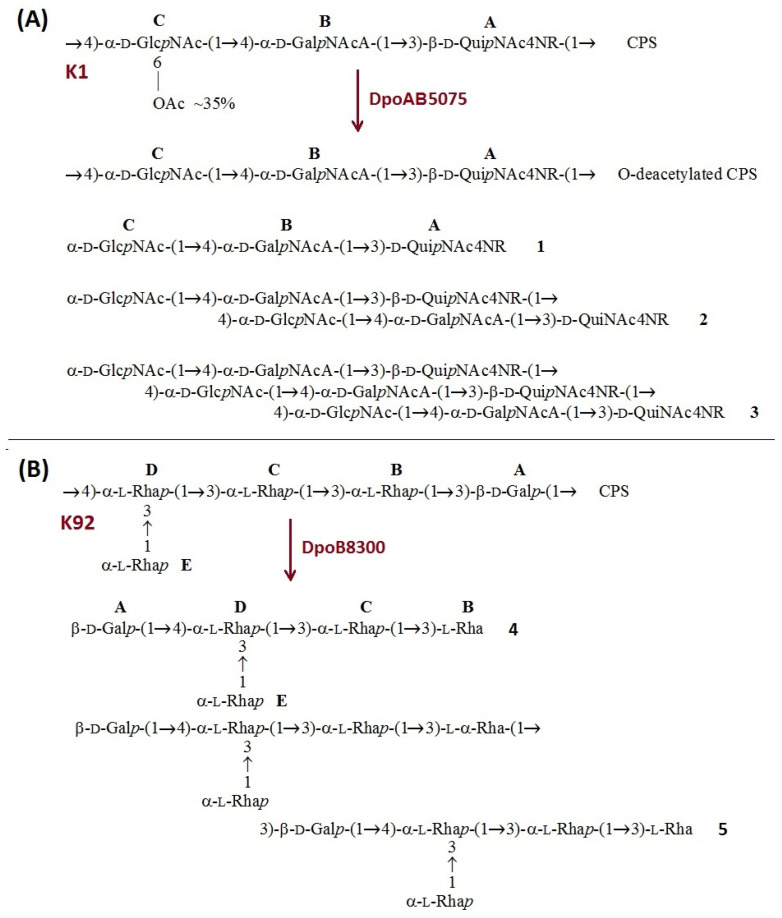
(**A**) Structures of the K1 CPS and O-deacetylated CPS from *A. baumannii* AYE and oligosaccharides **1–3** derived by depolymerization of the O-deacetylated CPS with prophage-derived depolymerase DpoAB5075. R = Ac or (S)-3-hydroxybutanoyl (~2:1). (**B**) Structures of the K92 CPS from *A. baumannii* B8300 and oligosaccharides **4** and **5** derived by depolymerization of the CPS with prophage-derived depolymerase DpoB8300.

The CPS of *A. baumannii* AYE has the same structure as the K1 CPS of *A. baumannii* 24 [16] and AB307-0294 [17]. It is composed of linear trisaccharide K units containing one residue each of 2-acetamido-4-acylamino-2,4,6-trideoxy-d-glucose (2-acetamido-4-acylamino-2,4-dideoxy-D-quinovose) (D-QuiNAc4NAcyl, where Acyl indicates acetyl or (S)-3-hydroxybutanoyl; units **A_Ac_** and **A_Hb_**, respectively), 2-acetamido-2-deoxy-D-galacturonic acid (D-GalNAcA, unit **B**), and N-acetyl-d-glucosamine (d-GlcNAc, unit **C**) (Figure 6A). The CPS is non-stoichiometrically (in ~35% K units) O-acetylated, and both intact and O-deacetylated CPSs were studied.

The K92 CPS of strain B8300 is composed of branched pentasaccharide K units containing one residue of β-D-Galp (unit **A**) and four residues of α-L-Rhap (units **B**–**E**) [18] (Figure 6B).

The CPSs were cleaved with recombinant prophage-derived depolymerases, and oligosaccharide products were fractionated by Fractogel TSK HW-40S gel-permeation chromatography. As a result, oligosaccharides **1–3** were obtained from the O-decatylated CPS from strain AYE upon cleavage by depolymerase DpoAB5075. The intact AYE CPS was cleaved with the depolymerase in a similar manner, giving rise to the corresponding non-stoichiometrically O-acetylated oligosaccharides (data not shown). Treatment of the B8300 CPS with depolymerase DpoB8300 resulted in oligosaccharides **4** and **5**.

The structures of the oligosaccharides obtained by depolymerization of the CPS were established by one- and two-dimensional ^1^H (Figures S2 and S3) and ^13^C NMR spectroscopy [19] and positive and negative ion modes high-resolution electrospray ionization mass spectrometry HR ESI MS (Figure S4) [20]. All oligosaccharides were found to have the same monosaccharide composition as the CPS they were derived from (Figure 6A,B).

The ^1^H and ^13^C NMR spectra of smaller oligosaccharides **1** and **4**, which corresponded to the K units of the CPSs, were fully assigned by two-dimensional shift-correlated experiments (^1^H-^1^H COSY, ^1^H-^1^H TOCSY, and ^1^H-^13^C HSQC) and compared with the data of the corresponding CPSs (Tables S2 and S3). Linkage and sequence analyses by two-dimensional ^1^H-^1^H ROESY and ^1^H-^13^C HMBC experiments enabled an elucidation of structures of **1** and **4**, which were confirmed by HR ESI MS (Table S4).

The ^1^H and ^13^C NMR spectra of larger oligosaccharides **2**, **3**, and **5** showed two series of signals, one series corresponding to smaller oligosaccharides **1** and **4** and the other to the CPS K units. Based on these and HR ESI MS data (Table S3), it was concluded that **2** and **5** corresponded to dimers and **3** to a trimer of the K units.

The data obtained indicated that oligosaccharides **1**–**5** were derived from the CPSs by specific hydrolytic cleavage of a linkage between the K units. Therefore, prophage-derived depolymerases DpoAB5075 and DpoB8300 are glycosidases that cleave the β-D-QuipNAc4NAcyl-(1→4)-D-GlcpNAc and α-L-Rhap-(1→3)-D-Galp linkages in the CPSs of *A. baumannii* AYE and B8300, respectively.

### 2.6. Galleria Mellonella Larvae Infection Model

**Figure 7:**
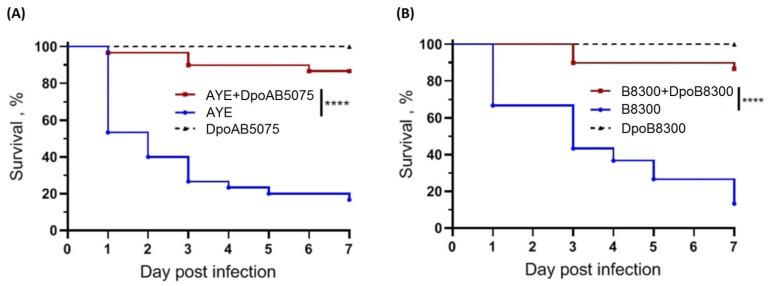
Kaplan–Meier survival curves following injection of *G. mellonella* larvae with *A. baumannii* strains treated and non-treated with depolymerases. (**A**) Larvae treatment with 1 × 10^6^ CFU of *A. baumannii* AYE; (**B**) Larvae treatment with 1 × 10^7^ CFU of *A. baumannii* B8300. Larvae (*n* = 30) were injected with either bacteria or bacteria simultaneously with the enzyme DpoAB5075 or DpoB8300 (2 μg per larva). The experiments were controlled by observation of uninfected larvae, larvae injected with saline solution, and larvae receiving the depolymerase only. Survival for each control group was 100%, so for simplicity, a group of uninfected larvae, and larvae injected with the saline solution, were not included in the figure. Statistically significant differences in survival between larvae infected with bacteria only and larvae infected with bacteria simultaneously with depolymerases were estimated by the log-rank (Mantel–Cox) test (**** *p* < 0.0001).

To evaluate the antivirulent potential of depolymerases, DpoAB5075 and DpoB8300, we used the *G. mellonella* larvae model of *A. baumannii* infection, induced by strains AYE and B8300. To determine larval susceptibility to infection with *A. baumannii* B8300 and AYE, we injected them with different inocula and monitored mortality daily. The doses of 1 × 10^7^ CFU and 1 × 10^6^ CFU for *A. baumannii* B8300 and AYE, respectively, were selected. In both cases, a gradual reduction in larval survival rates occurred over the 7-day experiments with that amount. At the end of the 7-day follow-up period, 86.7%, and 80.0% of the larvae died after inoculation with *A. baumannii* B8300 and AYE, respectively. At the same time, a single dose of the enzyme DpoB8300 or DpoAB5075 injected together with bacteria significantly inhibited *A. baumannii*-induced death in a time-dependent manner (Figure 7). Only a 13% mortality of larvae was recorded within 7 days after inoculation of *A. baumannii* B8300 together with depolymerase DpoB8300. Depolymerase DpoAB5075 also significantly increased the survival of larvae when co-administered with the strain *A. baumannii* AYE. In this case, the injection of 2 µg of depolymerase together with the infecting bacteria resulted in 80% survival of the larvae.

No mortality of larvae was observed in the controls, among uninfected larvae, larvae injected with saline solution, and larvae injected with the depolymerases. 

## 3. Discussion

In this study, six structural depolymerases encoded in different *A. baumannii* genomes were bioinformatically predicted, recombinantly produced, and studied. The examination of bacterial genomes, where the sequences were found, testified that they apparently belonged to prophage regions containing genes encoding phage assembly and structural proteins, products involved in phage nucleotide metabolism, the packaging of DNA into the capsid, bacterial cell lysis, and integration of phage DNA into bacterial genomes.

According to the BLASTp analysis, the identified depolymerases were identical or very closely homologous to various proteins encoded in other *A. baumannii* genomes. Considering the high prevalence of prophages in *A. baumannii* genomes [7,8], we assumed that prophage-derived structural depolymerases are widespread, and different bacterial genomes contain genes encoding proteins with polysaccharide-degrading activities.

Protein remote homology detection by HMM-HMM comparison conducted with HHpred demonstrated that the depolymerases possessed a multi-domain structure. The N-terminal parts of some depolymerases shared a similarity with the *Escherichia* phage T7 tail fiber. The remaining parts of all the proteins analysed contained the regions that showed similarities with different carbohydrate-hydrolyzing enzymes or lytic phage tailspikes. Thus, the studied proteins most likely assigned to prophage tailspike depolymerases, which participate in the first steps of interactions with bacterial hosts. Prediction of the domain organization of prophage-derived depolymerase monomers with AlphaFold 2 also revealed several regions corresponding to the N-terminal, central, and C-terminal parts.

Deletion mutants lacking the N-terminal domains responsible for the attachment of CPS-recognizing/degrading parts of the prophage-derived depolymerases to the phage particles were cloned, expressed, and purified. The strategy of cloning only receptor-binding/recognizing parts of the depolymerases was chosen in order to avoid possible protein aggregation due to the hydrophobicity of their N-termini. Moreover, we used this strategy in our previous studies on the characterization of tailspike depolymerases encoded in lytic phage genomes [21,22,23,24], where we showed the enzymatic activity of depolymerases without N-terminal parts towards corresponding CPSs.

The specificity of purified prophage-derived depolymerases was tested using a collection of *A. baumannii* strains belonging to 56 different capsular types. Two of six recombinant proteins expressed in *E. coli*, namely DpoAB5075 and DpoB8300, exhibited depolymerase activities on the bacterial lawns of *A. baumannii* AYE and B8300 belonging to K1 and K92 capsular types, respectively. Unfortunately, we were unable to determine the specificity of other purified prophage-derived depolymerases. Considering the fact that nowadays more than 144 K types were identified [5], this means that these proteins most likely could specifically interact with a CPS of one of those K types that were not tested in this study.

Taking into account that the CPSs are the primary receptors for depolymerase-carrying lytic *A. baumannii* phages [23], the first step of the infection of bacterial host by a temperate phage can also cause the degradation of corresponding CPS with a specific structural depolymerase. This is confirmed by the fact that prophage-derived depolymerase DpoB8300 degrades CPS of the K92 capsular type, to which the host strain *A. baumannii* B8300 also belongs. However, *A. baumannii* AB5075-UW, a representative of the K25 capsular type [25], encoded depolymerase DpoAB5075 which possess the enzymatic activity towards K1 CPS. This could be explained by possible recombination events between bacterial genomes or the horizontal transfer of prophage-containing regions.

The analysis of oligosaccharide products obtained by the degradation of the *A. baumannii* CPSs by recombinant prophage-derived depolymerases DpoAB5075 and DpoB8300 showed that the enzymes were specific glycosidases that cleaved the CPSs by the hydrolytic mechanism to produce a monomer and oligomers of the K1 and K92 units, respectively.

The capsule comprising repeating polysaccharide units is a major *A. baumannii* virulence determinant, which protects bacteria from host immune defences [17,26,27]. Thus, CPS-degrading enzymes or depolymerases encoded in phage genomes can represent effective antivirulence agents [28,29]. In recent years, the *Galleria mellonella* larvae were shown to be a reliable and cheap invertebrate model for studying the pathogenic mechanisms of microorganisms and the action of new antimicrobial agents [30]. The antivirulence efficacy of several depolymerases encoded in the genomes of lytic *A. baumannii*-phages was explored using a *G. mellonella* model [28,29]. In particular, a specific K2 depolymerase (B3gp42) encoding in the phage vB_AbaP_B3 genome (GenBank: MF033348) [28] was shown to protect larvae from bacterial infections, using either pretreatments or single-enzyme injections after bacterial challenges in a dose-dependent manner. In another study, depolymerase Dpo48 encoded in phage IME200 genome (GenBank: KT804908) was shown to reduce the virulence of *A. baumannii* host cells [29].

In this study, for the first time, we demonstrated that recombinant prophage-derived depolymerases with an established enzymatic activity could significantly reduce the mortality of *G. mellonella* larvae infected with *A. baumannii* of corresponding K types. In the long term, this means that specific prophage-derived depolymerases, as well as enzymes encoded in lytic phage genomes have great potential as antivirulence agents to control *A. baumannii* belonging to corresponding capsular types.

## 4. Materials and Methods

### 4.1. Bacterial Strains and Cultivation

*A. baumannii* strains B8300 and B11911 was kindly provided by Dr. Veeraraghavan Balaji (Christian Medical College, Vellore, India); strains AB2828, AB4932, AB5075-UW were kindly provided by Dr. D. Scholl (Pylum Biosciences, San Francisco, CA, USA); strain AYE was kindly provided by Dr. R. Zarrilli (University of Napoli Federico II, Naples, Italy); and strain NIPH60 was kindly provided by Dr. Alexandr Nemec (National Institute of Public Health, Prague, Czech Republic). 

The spectra of depolymerase activity of purified recombinant proteins were tested against a panel of *A. baumannii* strains with confirmed CPS structures (Table S1) belonging to different K types (K1, K2, K3, K6, K7, K8, K9, K11, K12, K15, K16, K17, K19, K20, K21, K24, K25, K27, K30, K32, K33, K35, K37, K42, K43, K44, K45, K46, K47, K48, K51, K52, K53, K54, K55, K57, K58, K61, K73, K74, K80, K81, K82, K83, K84, K85, K87, K88, K89, K90, K91, K92, K93, K116, K125, and K128).

The strains were provided courtesy of members of the *A. baumannii* research community (c.f., Acknowledgements). 

All bacteria were grown in Luria–Bertani (LB) broth (Difco, Detroit, MI, USA) or Nutrient agar (Himedia Laboratories Pvt. Limited, Mumbai, India) at 37 °C.

### 4.2. Bioinformatic Analysis

The bacterial genomic sequences were downloaded from GenBank [31]. Genomes of *A. baumannii* strains or contig sequences of interest were examined for potential prophage regions using PHASTER [9]. The open reading frames (ORFs) were validated and curated using a search of the NR (non-redundant) database of the NCBI [10] and a HHpred profile–profile search [11]. The homology search was performed by BLAST [32] using NCBI database and custom phage database with the E-value cut-off of 1 × 10^5^. Genetic maps were created using SnapGene software (from Insightful Science; available at snapgene.com, accessed on 12 January 2022). The tertiary structure prediction was made with AlphaFold 2.0 [13] with default settings and visualized with PyMOL (The PyMOL Molecular Graphics System, Version 2.0 Schrödinger, LLC.). The model quality assessment was performed with ModFOLD8 [33]. The alignments were made with Clustal Omega 1.2.3 [34,35] with the following settings: number of refinement iterations = 3, initial guide tree = evaluate full distance matrix, refinement iteration guide tree = evaluate full distance matrix, cluster size for mBed guide trees = 100. The alignments were trimmed manually. Best protein model was found with ModelTest-NG [36,37] integrated in raxmlGUI 2.0.7 graphic interface [38]. The phylogenetic tree was constructed with RAxML-NG [39] integrated in raxmlGUI 2.0.7 graphic interface using the PMB GAMMA F protein substitution model [40] and (ML + transfer bootstrap expectation + consensus) settings. The robustness of the RAxML-NG trees was assessed by bootstrapping (1000). The tree was visualized in Geneious Prime 2021.1 [41].

### 4.3. Cloning, Expression and Purification of the Recombinant Proteins

The DNA fragments of prophage-derived depolymerases lacking N-terminal domains were amplified by PCR using oligonucleotide primers, indicated in Table S5 and cloned into the the pTSL plasmid (GenBank accession KU314761) [42].

Expression vectors were transformed into chemically competent *E. coli* BL21(DE3) cells. Protein expression was performed in an LB medium supplemented with ampicillin at 100 mg/L. Transformed cells were grown at 37 °C until the optical density reached the value of 0.4 at 600 nm. The medium was cooled to the temperature of 16 °C followed by expression induction by an addition of isopropyl-1-thio-β-D-galactopyranoside (IPTG) to a final concentration of 1 mM. After further incubation at 16 °C overnight (approximately 16 h), the cells were harvested by centrifugation at 3700× *g* for 20 min, 4 °C. The cell pellets were resuspended in 1/50th of the original cell volume in buffer A (20 mM Tris pH 8.0, 0.5 M NaCl, 20 mM imidazole), complemented with 1 mg/mL lysozyme, and then lysed by sonication. The cell debris was removed by centrifugation at 16,000× *g* for 30 min, 4 °C. The supernatants were loaded onto nickel Ni^2+^-charged 5 mL GE HisTrap columns (GE Healthcare Life Sciences, Marlborough, MA, USA) equilibrated with buffer A containing 20 mM imidazole, and eluted with a 20–500 mM imidazole linear gradient in buffer A. The fractions containing the target proteins were pulled together and set up at 4 °C for the His-tag overnight digestion with TEV-protease at a protease/protein ratio of 1/100 (*w*/*w*). This reaction mixture was simultaneously dialyzed against 20 mM Tris pH 8.0, 200 mM NaCl, 0.5 mM DTT buffer resulting into His-SlyD expression tag removal. Protein samples after digestion were applied to the His-Trap column as before. A flow through concentrated with Sartorius ultrafiltration devices (molecular weight cutoff of 10,000) was applied to a Superdex 200 Hiload 16/60 column pre-equilibrated in 20 mM Tris-HCl, pH 7.5, and 150 mM NaCl (buffer B). The final protein samples were concentrated and stored in the same buffer at 4 °C.

### 4.4. Lawn Spot Assay

Enzymatic activity of prophage-derived depolymerases was tested by spotting protein solutions onto the bacterial lawns of different *A. baumannii* K types prepared using the double layer method [43]. For this, 300 μL of *A. baumannii* host strain cultures grown in LB medium at 37 °C to OD_600_ of 0.3 were mixed with 4 mL of soft agar (LB broth supplemented with 0.6% agarose). Mixture was plated onto nutrient agar. Then, 10 μL aliquots of solutions containing N-deletion mutants of depolymerases, and their tenfold dilutions were spotted on the soft agar lawns and incubated at 37 °C for 18–24 h.

### 4.5. Isolation and Purification of the CPSs

Bacteria were cultivated in 2TY media (16 g Bacto Tryptone, 10 g Bacto Yeast Extract, and 5 g NaCl, adjusted to 1 L with distilled H_2_O) for 16 h. Cells were harvested by centrifugation (10,000× *g*, 20 min), washed with phosphate-buffered saline, suspended in aqueous 70% acetone, precipitated, and dried on air. Capsular polysaccharides were isolated by extraction of bacterial cells of *A. baumannii* AYE and B8300 with 45% aqueous phenol for 30 min at 65–68 °C [44]. The extract was cooled, dialyzed without layer separation, freed from insoluble contaminations by centrifugation (12,000× *g*, 20 min), and CPS preparations were purified as described [45]. Briefly, aqueous 50% CCl_3_CO_2_H was added to a CPS solution in water at 4 °C, a precipitate was removed by centrifugation, the supernatant was dialyzed against distilled water and freeze-dried. To cleave the accompanying short-chain lipopolysaccharide, the CPS preparations were heated with 2% HOAc (100 °C, 3 h), and a lipid precipitate was removed by centrifugation (12,000× *g*, 20 min). Purified CPS samples were isolated from the supernatant by gel-permeation chromatography on a XK 26/70 column (700 × 26 mm, gel layer 560 mm) (GE Healthcare, Pollards Wood, UK) of Sephadex G-50 Superfine (Amersham Biosciences, Uppsala, Sweden) in 0.05 M pyridinium acetate buffer pH 4.5. Flow rate was 0.5 mL/min; elution was monitored with a differential refractometer (Knauer, Berlin, Germany). Control of retention of the intact structure upon mild acid treatment was performed by NMR spectroscopy. A CPS sample from strain AYE was treated with 12.5% aqueous ammonia (60 °C, 3 h), ammonia was removed by stream of air, and an O-deacetylated CPS sample was obtained by liophylization of the remaining solution.

### 4.6. Cleavage of the CPSs with Prophage-Derived Depolymerases

Purified CPSs were solubilized at the 20 mM Tris-HCl pH7.5, and 500 µg of recombinant proteins were added for digestion. The reaction mixture was incubated at 37 °C overnight.

CPS digestion products were fractionated by gel-permeation chromatography on a XK 16/100 column (110 cm × 16 mm, gel layer 80 cm) (GE Healthcare, Pollards Wood, UK) of Fractogel TSK HW-40S (Toyo Soda, Tokyo, Japan) in 1% acetic acid at a flow rate 0.5 mL/min monitored as above.

### 4.7. NMR Spectroscopy

Samples were deuterium-exchanged by freeze-drying from 99.9% D_2_O and then examined as the solution in 99.95% D_2_O. NMR spectra were recorded on a Bruker Avance II 600 MHz spectrometer (Bruker, Bremen, Germany) at 30–60 °C. Sodium 3-trimethylsilylpropanoate-2,2,3,3-d_4_ (δ_H_ 0, δ_C_ –1.6) was used as internal reference for calibration. Two-dimensional ^1^H-^1^H correlation spectroscopy (COSY), ^1^H-^1^H total correlation spectroscopy (TOCSY), ^1^H-^1^H rotating-frame nuclear Overhauser effect spectroscopy (ROESY), ^1^H-^13^C heteronuclear single-quantum coherence (HSQC), and ^1^H-^13^C heteronuclear multiple-bond correlation (HMBC) experiments were performed using standard Bruker software and used for assignment of ^1^H and ^13^C NMR chemical shifts [19]. Bruker TopSpin 2.1 program was used to acquire and process the NMR data. A MLEV-17 spin-lock time of 60 ms and a mixing time of 200 ms were used in TOCSY and ROESY experiments, respectively. A 60 ms delay was used for the evolution of long-range couplings to optimize the ^1^H-^13^C HMBC experiment for coupling constant *J*_H,C_ 8 Hz.

### 4.8. Mass Spectrometry

High-resolution electrospray ionization mass spectrometry (HR ESI MS) [20] was performed in positive and negative ion modes using a micrOTOF II or maXis instruments (Bruker Daltonics, Bremen, Germany). Oligosaccharide samples (~50 ng/µL) were dissolved in a 1:1 (*v*/*v*) water/acetonitrile mixture and injected with a syringe at a flow rate of 3 µL/min. Capillary entrance voltage was set at –4500 V (positive ion mode) or 3000 V (negative ion mode), and the interface temperature at 180 °C. Nitrogen was used as the drying and sheath gas. Mass range was set from *m/z* 50 to 3000. Internal calibration was conducted with ESI Tuning Mix (Agilent, Santa Clara, CA, USA).

### 4.9. Galleria Mellonella Larvae Infection Model

Culture and infection of *G. mellonella* larvae with *A. baumannii* strains and the estimation of the survival rate of infected larvae were performed as previously described [46]. Briefly, larvae were infected by injection into the hemocoel with 1 × 10^7^ CFU (*A. baumannii* B8300) or 1 × 10^6^ CFU (*A. baumannii* AYE) of (i) bacteria only, and (ii) bacteria administered together with the depolymerase (2 μg per larvae). Three control groups were used: uninfected larvae, larvae injected with saline solution, and larvae injected with depolymerase. Infected larvae were incubated at 37 °C for 7 days and mortality was recorded daily. Each test was performed in triplicate, with 10 larvae per trial. The GraphPad Prism software (GraphPad Software, Inc., La Jolla, CA, USA) was used for statistical analysis and graphical presentation of the results. Statistical analysis was performed for pairwise comparisons between larvae infected with bacteria only and larvae infected with bacteria simultaneously with depolymerase using log-rank (Mantel-Cox) test. Values of *p* < 0.05 were considered statistically significant.

## Figures and Tables

**Table 1:** Prophage-derived depolymerases identified in *A. baumannii* genomes.

Depolymerase Designation	*A. baumannii* Strain/GenBank Accession Number of Genome or Contig Sequence ^1^	Locus/Coordinates in Genome or Contig	Gene Product ^2^	GenBank Accession Number (Protein_Id)	Protein Size, aa
DpoAB2828	AB2828/ LRDT01000031	LV35_02359/ complement 78922–81369	hypothetical protein	KZA15926	815
DpoAB5075	AB5075-UW/ CP008706	ABUW_0568/ 566943–568895	phage tail fibre protein	AKA30338	650
DpoB8300	B8300/ CP021347	AB987_0146/ complement 142633–144540	hypothetical protein	KMV24774	635
DpoB11911	B11911/ CP021345	AB994_2560/ 2694400–2697699	carbohydrate binding domain protein	KMV03800	1099
DpoAB4932	AB4932/ LREK01000014	LV53_01598/ 23828–25882	hypothetical protein	KZA74345	684
DpoNIPH60	NIPH60/APPM01000011	F961_00657/ 112235–114493	hypothetical protein	ENV30868	752

^1^ in which the prophage-derived depolymerases were identified ^2^ according to the annotation made by the authors of a sequence.

## Supplementary Materials

The following are available online at https://www.mdpi.com/article/10.3390/ijms23094971/s1.
