# Supplementary material for: Capsule-Targeting Depolymerases Derived from Acinetobacter baumannii Prophage Regions
Source: Int J Mol Sci. 2022 Apr 29;23(9):4971. doi: 10.3390/ijms23094971 (PMC9102105; doi:10.3390/ijms23094971)
Supplement: Supplementary file 1 [file ijms-23-04971-s001.zip › ijms-1688537-supplementary.pdf]

Supplementary Materials

Figure S1. Examples of 12% SDS-PAGE gels of the purified recombinant depolymerases lacking N-terminal domains.

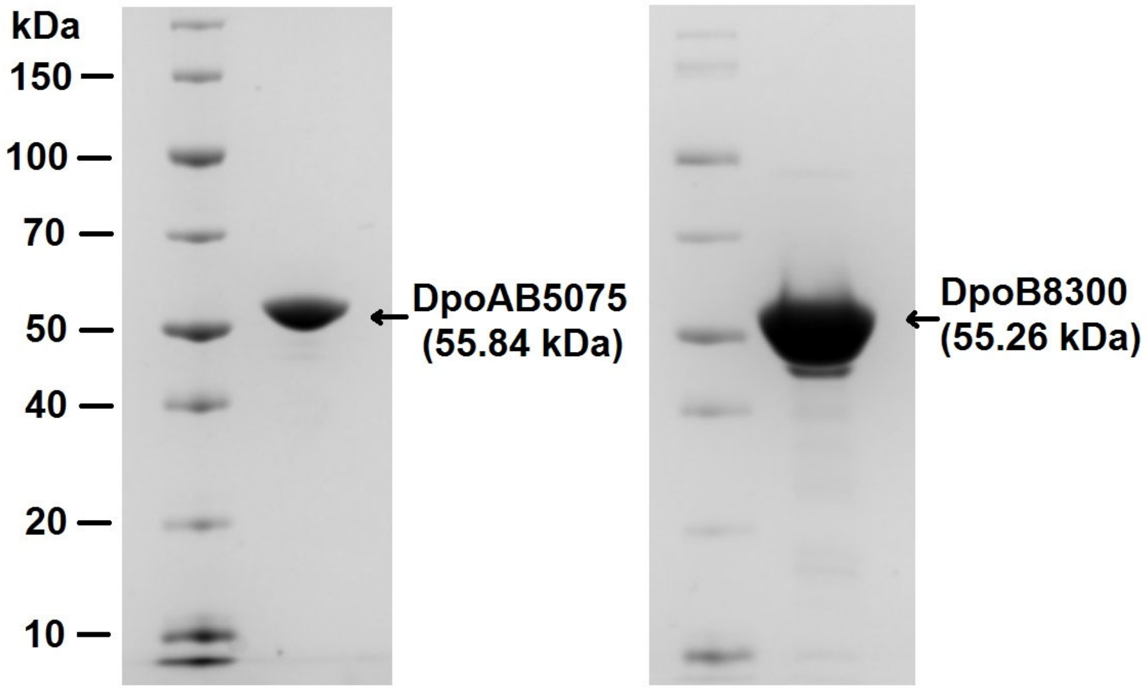

Figure S2. ^{1}H NMR spectrum of oligosaccharide **1**.

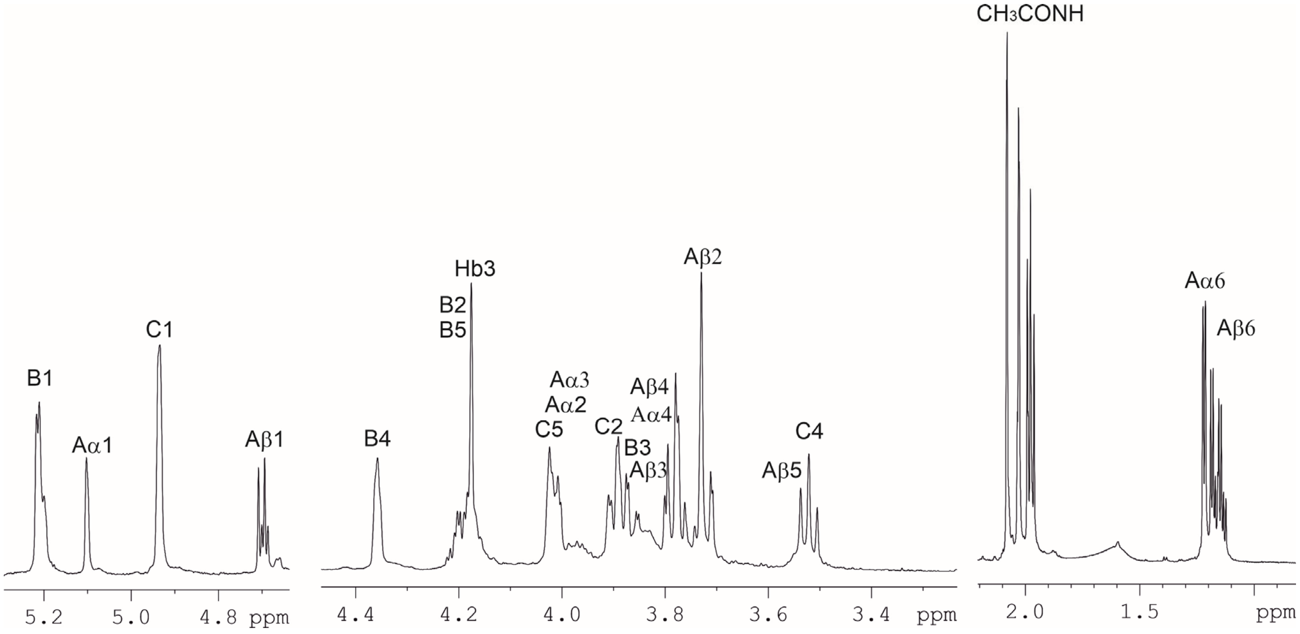

Figure S3. ^{1}H NMR spectrum of oligosaccharide **4**.

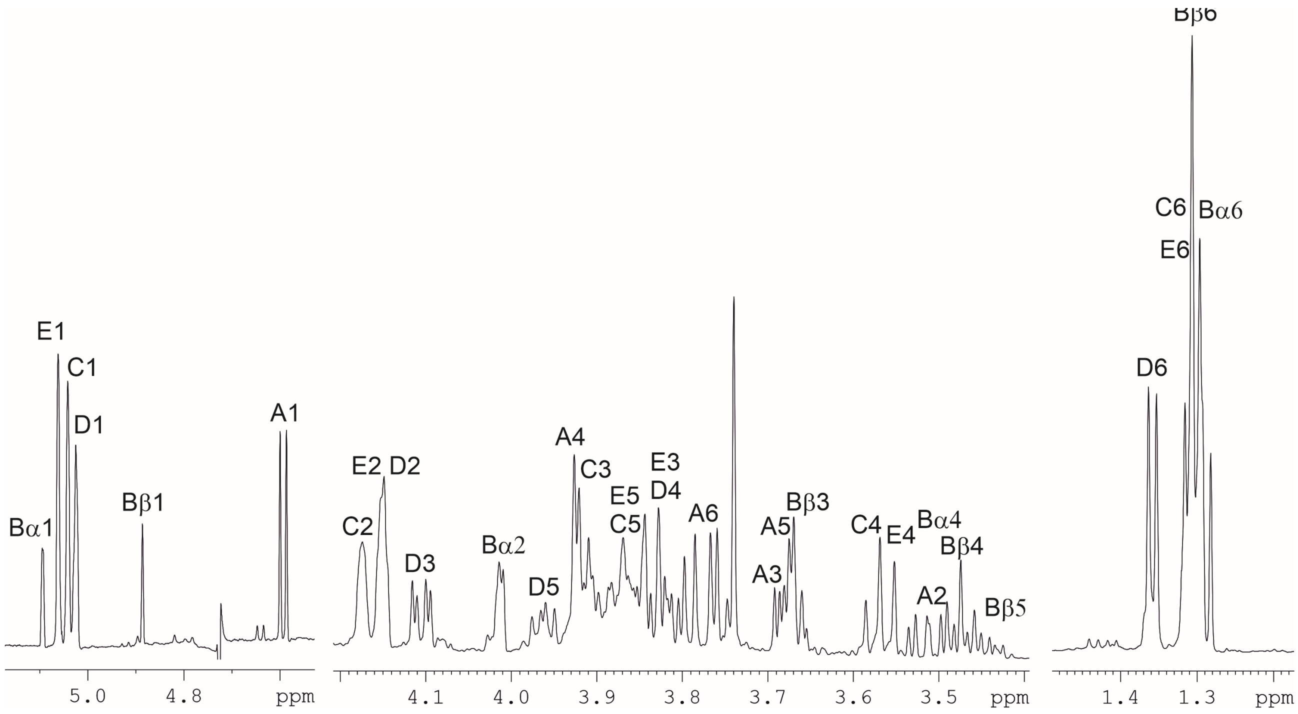

Figure S4. High-resolution ESI mass spectra of oligosaccharides **1—5**. **a, 1**$_{Ac}$ and **1**$_{Hb}$; **b, 2**$_{AcAc}$, **2**$_{AcHb}$, and **2**$_{HbHb}$, **c1, 3**$_{AcAcAc}$, **c2, 3**$_{AcAcHb}$, **c3, 3**$_{AcHbHb}$, **c4, 3**$_{HbHbHb}$, **d.** Hex_1dHex_4, **e.** Hex_2dHex_8

a

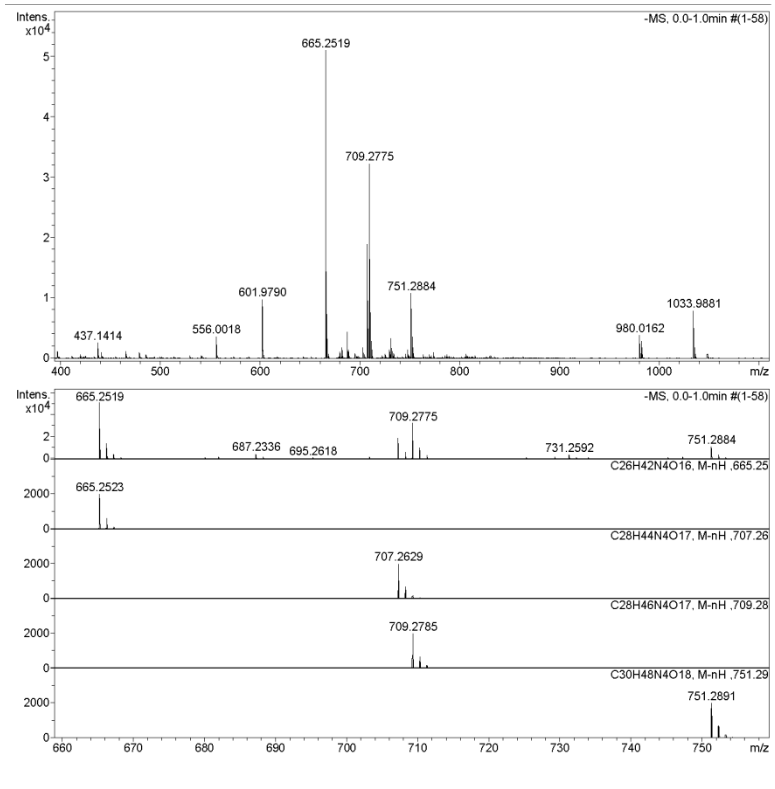

b

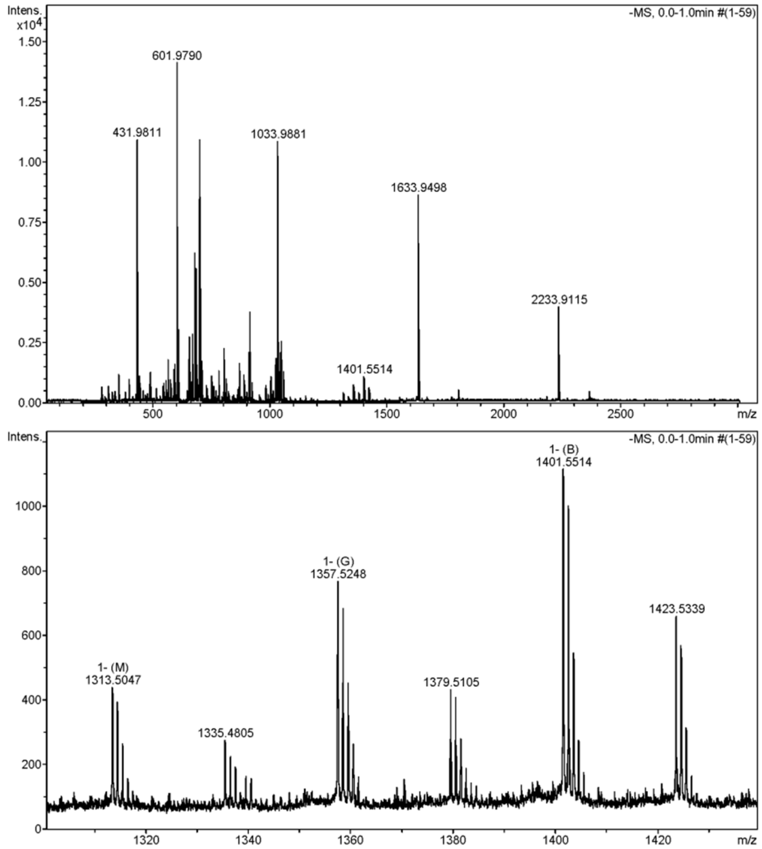
Intens.
x10^4
-MS, 0.0-1.0min #(1-59)
1.50
1.25
1.00
0.75
0.50
0.25
0.00
601.9790
431.9811
1033.9881
1633.9498
2233.9115
1401.5514
500
1000
1500
2000
2500
m/z
Intens.
-MS, 0.0-1.0min #(1-59)
1000
800
600
400
200
0
1- (B)
1401.5514
1- (G)
1357.5248
1423.5339
1- (M)
1313.5047
1379.5105
1335.4805
1320
1340
1360
1380
1400
1420
m/z

c-1

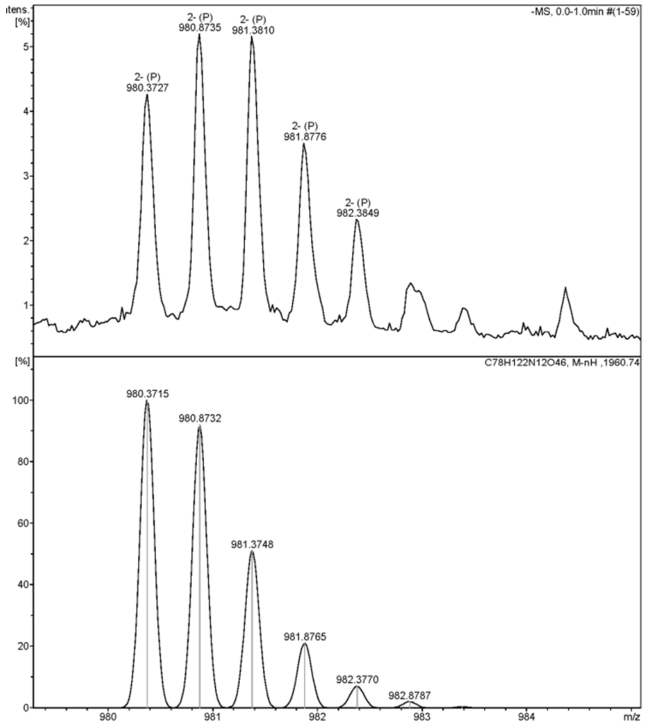
Intens.
[%]
-MS, 0.0-1.0min #(1-59)
5
4
3
2
1
2- (P)
980.8735
2- (P)
981.3810
2- (P)
980.3727
2- (P)
981.8776
2- (P)
982.3849
[%]
C78H122N12O46, M-nH ,1960.74
100
80
60
40
20
0
980.3715
980.8732
981.3748
981.8765
982.3770
982.8787
980
981
982
983
984
m/z

c-2

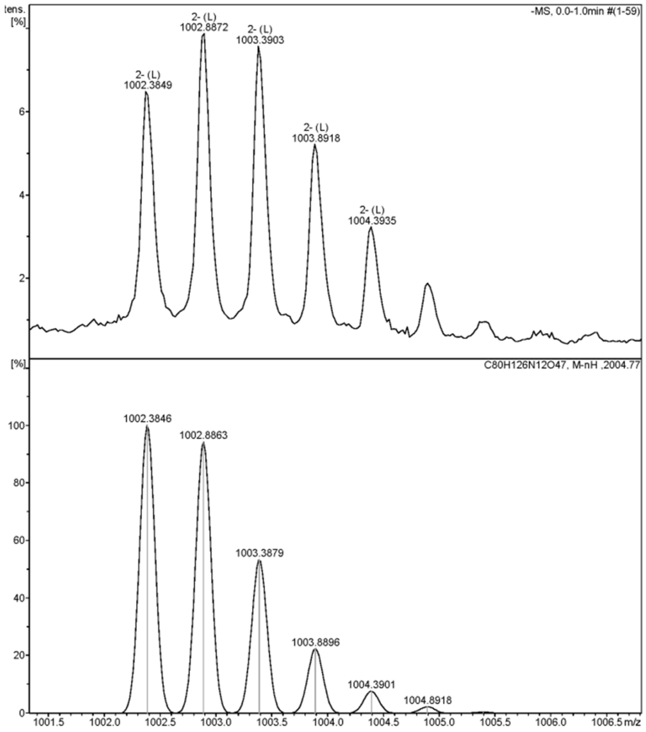

Intens. [%]
-MS, 0.0-1.0min #(1-59)
2- (L) 1002.3849
2- (L) 1002.8872
2- (L) 1003.3903
2- (L) 1003.8918
2- (L) 1004.3935
[%]
C80H126N12O47, M-nH ,2004.77
1002.3846
1002.8863
1003.3879
1003.8896
1004.3901
1004.8918
m/z

c-3

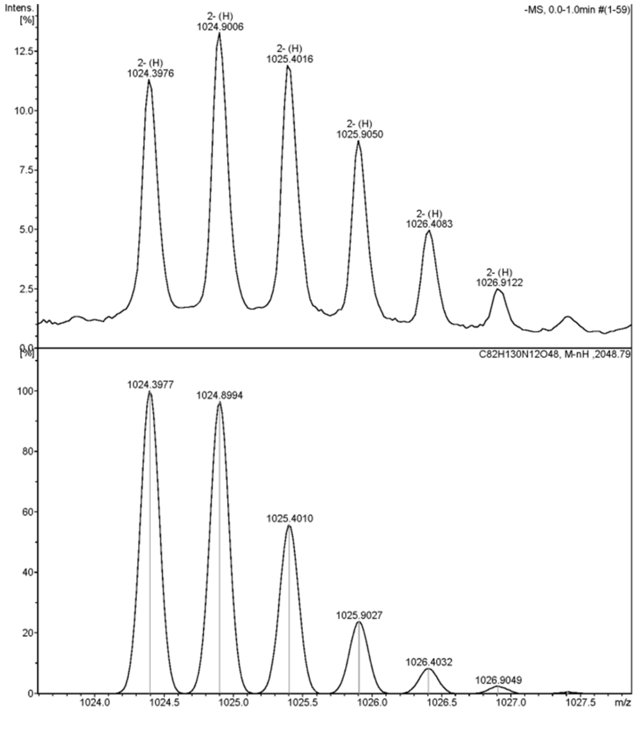

Intens. [%]
-MS, 0.0-1.0min #(1-59)
2- (H) 1024.3976
2- (H) 1024.9006
2- (H) 1025.4016
2- (H) 1025.9050
2- (H) 1026.4083
2- (H) 1026.9122
[%]
C82H130N12O48, M-nH ,2048.79
1024.3977
1024.8994
1025.4010
1025.9027
1026.4032
1026.9049
m/z

c-4

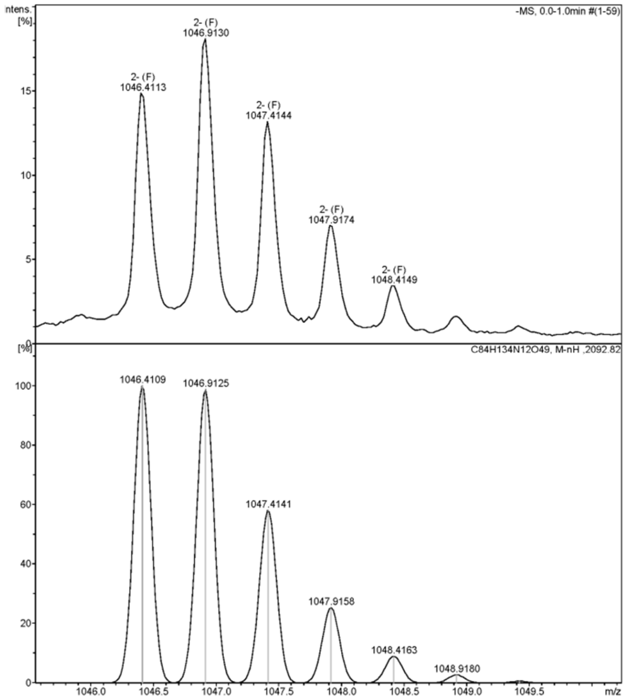
Intens. [%]
-MS, 0.0-1.0min #(1-59)
2- (F) 1046.4113
2- (F) 1046.9130
2- (F) 1047.4144
2- (F) 1047.9174
2- (F) 1048.4149
C84H134N12O49, M-nH ,2092.82
1046.4109
1046.9125
1047.4141
1047.9158
1048.4163
1048.9180
m/z

d

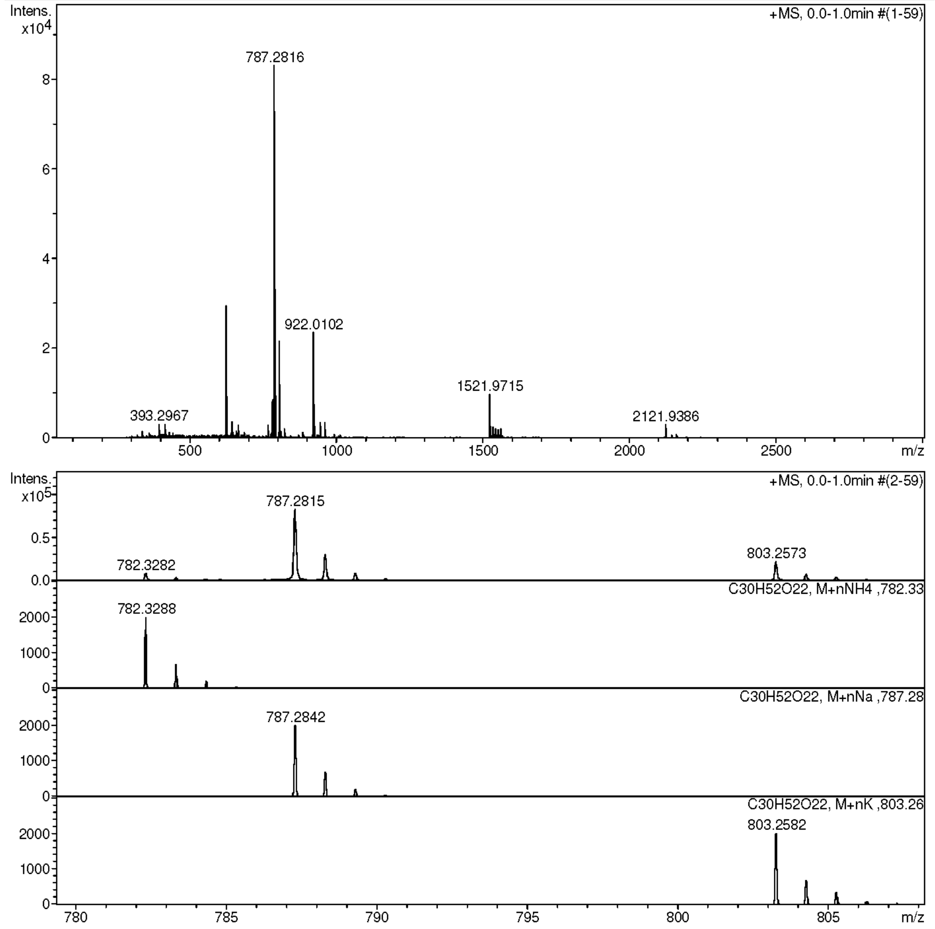
Intens. x10^4
+MS, 0.0-1.0min #(1-59)
787.2816
922.0102
393.2967
1521.9715
2121.9386
m/z
Intens. x10^5
+MS, 0.0-1.0min #(2-59)
787.2815
782.3282
803.2573
C30H52O22, M+nNH4 ,782.33
782.3288
C30H52O22, M+nNa ,787.28
787.2842
C30H52O22, M+nK ,803.26
803.2582
m/z

e

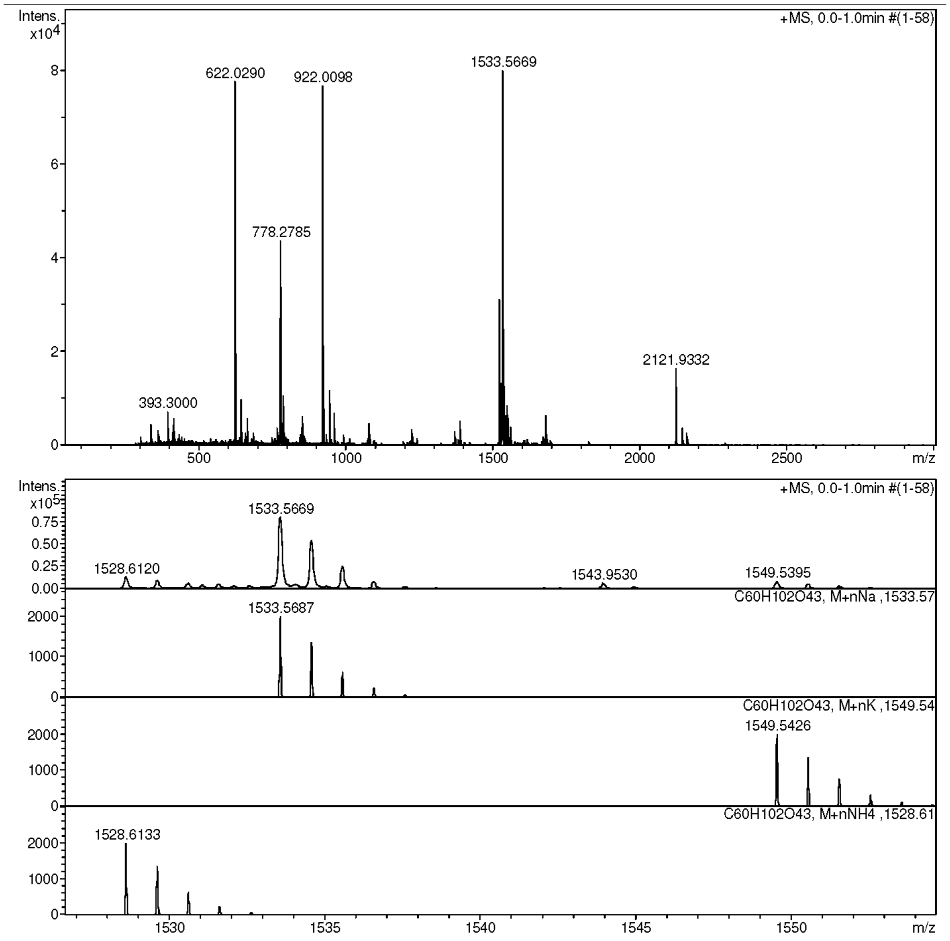

Table S1. *Acinetobacter baumannii* strains used in this study for the determination of specificity of prophage-derived depolymerases.

K type	*A. baumannii* strains
1	AYE
2	ACICU
3	AB4932
6	RBH4
7	LUH5533
8	BAL097
9	B05
11	LUH5545
12	AB2828
15	LUH5554
16	D4
17	G7
19	28

20	A388
21	G21
24	RCH51
25	AB5075
27	4190
30	NIPH190
32	LUH5549
33	NIPH67
35	LUH5535
37	NIPH146
42	LUH5550
43	NIPH60
44	NIPH70
45	NIPH201
46	NIPH329
47	NIPH601
48	NIPH615
51	WM98b
52	LUH5546
53	D23
54	RCH52
55	BAL204
57	BAL212
58	BAL114
61	NL4
73	SGH0703
74	BAL309
80	LUH3712
81	LUH3713
82	LUH5534
83	LUH5538
84	LUH5540
85	LUH5543
87	LUH5547
88	LUH5548
89	LUH5552
90	LUH5553
91	1053
92	B8300
93	B11911
116	MAR-303
125	MAR13-1452
128	KZ-1093

Table S2. ^{1}H and ^{13}C NMR chemical shifts of the O-deacetylated K1 CPS from *A. baumannii* AYE and oligosaccharide **1** derived by depolymerization of the O-deacetylated CPS with prophage-derived depolymerase DpoAB5075 (δ, ppm). Given are data for the major K unit of the O-deacetylated CPS and oligosaccharide 1_{Ac} containing QuiNAc4NAc.

Sugar residue	*C-1*<br>H-1	*C-2*<br>H-2	*C-3*<br>H-3	*C-4*<br>H-4	*C-5*<br>H-5	*C-6*<br>H-6 (6a,6b)
O-Deacetylated CPS (at 30 °C)						
→3)-β-D-QuipNAc4NAc	*101.6*	*56.2*	*76.6*	*58.1*	*72.7*	*18.0*
**A**	4.62	3.76	4.03	3.86	3.54	1.20
→4)-α-D-GalpNAcA-(1→	*98.7*	*50.6*	*67.7*	*80.0*	*72.9*	*175.2*
**B**	5.21	4.16	3.91	4.34	4.09	
→4)-α-D-GlcpNAc-(1→	*99.8*	*54.9*	*70.6*	*80.0*	*72.0*	*61.0*
**C**	4.93	4.15	3.90	3.67	4.08	3.63, 3.70
Oligosaccharide $\mathbf{1}_{Ac}$ (at 30 °C)						
→3)-α-D-Qui*p*NAc4Ac	*92.2*	*54.3*	*74.6*	*58.1*	*68.0*	18.0
$\mathbf{A\alpha}_{Ac}$	5.10	4.02	4.02	3.79	3.96	1.19
→3)-β-D-Qui*p*NAc4NAc	95.8	*57.3*	*76.8*	*58.0*	*72.4*	18.0
$\mathbf{A\beta}_{Ac}$	4.70	3.73	3.87	3.83	3.53	1.15
→4)-α-D-Gal*p*NAcA-(1→	*98.5*	*50.5*	*68.3*	*80.1*	*72.6*	174.6
**B**	5.21	4.20	3.88	4.36	4.17	
α-D-Glc*p*NAc-(1→	*100.3*	*55.0*	*72.2*	*70.8*	*73.6*	*61.3*
**C**	4.93	3.90	3.78	3.52	4.01	3.72, 3.78

^{13}C NMR chemical shifts are italicized.
Signals for N-acetyl groups are at δ_C 23.2-23.7 (Me) and 175.3-175.9 (CO), δ_H 1.96-2.08.

Table S3. ^{1}H and ^{13}C NMR chemical shifts of the K92 CPS of *A. baumannii* B8300 and oligosaccharide **4** derived by depolymerization with prophage-derived depolymerase DpoB8300 (δ, ppm)

Sugar residue	*C-1* H-1	*C-2* H-2	*C-3* H-3	*C-4* H-4	*C-5* H-5	C-6 H-6 (6a,6b)
CPS (at 60 °C)						
→3)-β-D-Gal*p*-(1→	*104.2*	*71.7*	*81.8*	*69.7*	*76.1*	*62.1*
**A**	4.63	3.65	3.71	4.04	3.67	3.76, 3.81
→3)-α-L-Rha*p*-(1→	*103.3*	*71.2*	*79.7*	*72.6*	*70.5*[a]	*17.9*[b]
**B**	5.08	4.17	3.92	3.59	3.88	1.29
→3)-α-L-Rha*p*-(1→	*103.2*	*71.3*[c]	*79.4*	*72.6*	*70.6*[a]	*17.9*[b]
**C**	5.06	4.14	3.92	3.59	3.88	1.30
→3,4)-α-L-Rha*p*-(1→	*103.1*	*71.5*[c]	*80.5*	*78.4*	*69.3*	*18.3*
**D**	5.05	4.14	4.10	3.86	3.96	1.36
α-L-Rha*p*-(1→	*103.6*	*71.2*	*71.6*	*73.4*	*70.4*	*17.8*[b]
**E**	5.08	4.17	3.85	3.48	3.86	1.29
Oligosaccharide **4** (at 30 °C)						
→3)-α-L-Rha*p*	*95.2*	*71.9*	*79.1*	*73.3*	*70.5*	*18.1*
**Bα**	5.09	4.01	3.85	3.47	3.91	1.29
→3)-β-L-Rha*p*	*94.6*	*72.5*	*81.5*	*73.3*	*73.2*	*17.9*
**Bβ**	4.88	4.01	3.66	3.47	3.44	1.31
→3)-α-L-Rha*p*-(1→	*103.4*	*71.2*	*79.9*	*72.5*	*70.5*	*17.8*[c]
**C**	5.04	4.17	3.92	3.57	3.87	1.30
→3,4)-α-L-Rha*p*-(1→	*103.4*	*71.4*	*80.9*	*78.3*	*69.4*	*18.4*
**D**	5.03	4.14	4.10	3.83	3.96	1.36
β-D-Gal*p*-(1→	*104.5*	*72.2*	*74.0*	*70.0*	*76.2*	*62.3*
**A**	4.59	3.51	3.68	3.92	3.67	3.76, 3.79
α-L-Rha*p*-(1→	*103.9*	*71.3*	*71.5*	*72.8*	*70.5*	*17.9*[c]
**E**	5.06	4.15	3.83	3.55	3.87	1.30

^{13}C NMR chemical shifts are italicized.
[a-c]Assignment could be interchanged

Table S4. HR ESI MS data of oligosaccharides **1-5**.

Oligosaccharide	Composition	Molecular mass, Da	Ion peak at *m/z* (experimental/*calculated*)				
			$[M-H]^-$	$[M+H]^+$	$[M+NH_4]^+$	$[M+Na]^+$	$[M+K]^+$
**1**$_{Ac}$	$HexN_1HexNA_1dHexNN_1Ac_4$	666.2596	665.2519/ 665.2523				
**1**$_{Hb}$	$HexN_1HexNA_1dHexNN_1Ac_3Hb_1$	710.2858	709.2775/ 709.2785				
**2**$_{AcAc}$	$HexN_2HexNA_2dHexNN_2Ac_8$	1314.5086	1313.5047/ *1313.5013*				
**2**$_{AcHb}$	$HexN_2HexNA_2dHexNN_2Ac_7Hb_1$	1358.5348	1357.5248/ *1357.5275*				
**2**$_{HbHb}$	$HexN_2HexNA_2dHexNN_2Ac_6Hb_2$	1402.5610	1401.5514/ *1402.5538*				
**3**$_{AcAcAc}$	$HexN_3HexNA_3dHexNN_3Ac_{12}$	1962.7576	980.3727/ *980.3715*[a]				
**3**$_{AcAcHb}$	$HexN_3HexNA_3dHexNN_3Ac_{11}Hb_1$	2006.7838	1002.3849/ *1002.3846*[a]				
**3**$_{AcHbHb}$	$HexN_3HexNA_3dHexNN_3Ac_{10}Hb_2$	2050.8101	1024.3976/ *1024.3977*[a]				
**3**$_{HbHbHb}$	$HexN_3HexNA_3dHexNN_3Ac_9Hb_3$	2094.8363	1046.4113/ *1046.4109*[a]				
**4**	Hex_1dHex_4	764.2950			782.3277/ *782.3288*	787.2810/ *787.2842*	803.2568/ *803.2582*
**5**	Hex_2dHex_8	1510.5794			1528.6120/ *1528.6133*	1533.5669/ *1533.5687*	1549.5395/ *1549.5428*

Hex, dHex, HexN, HexNA, dHexNN indicate hexose, deoxy hexose, aminodeoxy hexose, aminodeoxy hexuronic acid, diaminotrideoxy hexose, respectively; Hb indicates hydroxybutanoyl.

[a]Data for an $[M-2H]^{2-}$ ion.

Table S5. Oligonucleotide primers used in this study for cloning of prophage-derived depolymerases.

Primer	Sequence (5′–3′)	Restriction site for:
AB2828_F	ataGGATCCaataacaacaccaccgatccg	BamHI
AB2828 _R	tatCTCGAGttaactaatacctagcaagttgaag	XhoI
AB5075_F	ataGGATCCgaagaagccaaactagaagc	BamHI
AB5075 _R	ataAAGCttataaaaagactgaaactttcca	HindIII
B8300_F	ataGGATCCtcagcggatgcttctttcga	BamHI
B8300 _R	ataCTCGAGttaactaaagacataccagcct	XhoI
B11911_F	ataGGATCCgaggatgcaaaagaagaagca	BamHI
B11911 _R	ataCTCGAGttacaaggcggccaagataaa	XhoI
NIPH60_F	ataGGATCCgatggcgatgagagtcaaaaa	BamHI
NIPH60 _R	ataCTCGAGttacatgttgcttaataatagacg	XhoI
AB4932_F	ata GGATCCagtgcagccggagcaggc	BamHI
AB4932 _R	ataCTCGAGttaaattcctgattctaatgtgaa	XhoI

Uppercase letters indicate restriction endonuclease recognition sites.
